# Anti-Cancer Effects of a New Herbal Medicine PSY by Inhibiting the STAT3 Signaling Pathway in Colorectal Cancer Cells and Its Phytochemical Analysis

**DOI:** 10.3390/ijms232314826

**Published:** 2022-11-27

**Authors:** Sanghee Han, Hail Kim, Min Young Lee, Junhee Lee, Kwang Seok Ahn, In Jin Ha, Seok-Geun Lee

**Affiliations:** 1 Graduate School, Kyung Hee University, Seoul 02447, Republic of Korea; 2 Korean Medicine Clinical Trial Center, Kyung Hee University Korean Medicine Hospital, Seoul 02454, Republic of Korea; 3 BioNanocomposite Research Center, Kyung Hee University, Seoul 02447, Republic of Korea

**Keywords:** Patriniae Radix, Mori Cortex Radicis, Coix Seed, PSY, colorectal cancer, STAT3

## Abstract

Colorectal cancer (CRC) is an inflammation-associated common cancer worldwide. Paejang-san and Mori Cortex Radicis have been traditionally used for treating intestinal inflammatory diseases in Korea and China. In the present study, we developed a new herbal formula as an alternative to CRC treatments, which is composed of two main components of Paejangsan (Patriniae Radix (Paejang in Korean) and Coix Seed (Yiyiin in Korean)), and Mori Cortex Radicis (Sangbekpi in Korean) based on the addition and subtraction theory in traditional medicine, hence the name PSY, and explored the potential therapeutic effects of the new formula PSY in human CRC cells by analyzing viability, cell cycle and apoptosis. We found that PSY ethanol extract (EtOH-Ex), but not water extract, significantly suppressed the viability of human CRC cells, and synergistically decreased the cell proliferation compared to each treatment of Patriniae Radix and Coix Seed extract (PY) or Mori Cortex Radicis extract (S), suggesting the combination of PY and S in a 10-to-3 ratio for the formula PSY. PSY EtOH-Ex in the combination ratio reduced cell viability but induced cell cycle arrest at the G_2_/M and sub-G_1_ phases as well as apoptosis in CRC cells. In addition, the experimental results of Western blotting, immunofluorescence staining and reporter assays showed that PSY also inhibited STAT3 by reducing its phosphorylation and nuclear localization, which resulted in lowering STAT3-mediated transcriptional activation. In addition, PSY regulated upstream signaling molecules of STAT3 by inactivating JAK2 and Src and increasing SHP1. Moreover, the chemical profiles of PSY from UPLC-ESI-QTOF MS/MS analysis revealed 38 phytochemicals, including seven organic acids, eight iridoids, two lignans, twelve prenylflavonoids, eight fatty acids, and one carbohydrate. Furthermore, 21 potentially bioactive compounds were highly enriched in the PSY EtOH-Ex compared to the water extract. Together, these results indicate that PSY suppresses the proliferation of CRC cells by inhibiting the STAT3 signaling pathway, suggesting PSY as a potential therapeutic agent for treating CRC and 21 EtOH-Ex-enriched phytochemicals as anti-cancer drug candidates which may act by inhibiting STAT3.

## 1. Introduction

Colorectal cancer (CRC) is the third most common cancer in the world, the third most diagnosed cancer among men, and the second most common among women. Overall, CRC ranks third in terms of incidence but second in terms of mortality [1]. Incidence rates have steadily risen in many countries in eastern Europe, southeastern and south-central Asia, and South America. Risk factors include the consumption of red or processed meat and heavy alcohol consumption, whereas adequate consumption of whole grains, fiber, and dairy products decreases the risk [2]. Primary prevention remains a key strategy for reducing the increasing global burden of CRC. The largest proportion of CRC cases has been linked to environmental mutations rather than heritable genetics [2]. Inflammatory bowel disease (IBD) is an important risk factor for colon cancer. In this regard, colitis-associated cancer is a CRC subtype associated with IBD, is challenging to treat, and has high mortality [3]. Moreover, CRC exhibits constitutive activation of NF-κB and STAT3, transcription factors that influence interactions between tumor cells and tumor microenvironment and play integrated roles in cancer-promoting inflammation [4]. Further, inflammation is associated with tumor cell proliferation, survival, metastasis, angiogenesis, and chemoresistance and may also affect the efficacy of CRC therapies, including STAT3 inhibitors [4,5]. However, NF-κB is also involved in anti-tumor immune responses, and in contrast, STAT3 restrains the NF-κB-mediated anti-tumor immunity [4]. Thus, STAT3 has been suggested as a more promising target for cancer therapy by redirecting inflammation [4,5]. Recent studies have also indicated that CRC progression occurs by activating tumorigenic JAK/STAT3 signaling [5,6].

Although many cancer medications such as oxaliplatin for treating CRC are available, better options have been requested for cancer patients because of the low efficacy and severe side effects of conventional chemotherapies [7,8]. Thus, medicinal plants and dietary phytochemicals have attracted great attention due to strong beliefs that as these substances are edible they have minimal toxicity [9].

Roots of *Patrinia scabiosaefolia* Fisch. (Patriniae Radix (PR)) and seeds of *Coix lacryma-jobi* L. var. *ma-yuen* Stapf (Coix Seed (CS)) are the main components of Paejangsan which has been traditionally used to treat intestinal inflammatory diseases in Korea and China [10]. The root bark of *Morus alba* L. (Mori Cortex Radicis (MCR)) has been used for inflammation-related diseases in the intestine and lungs in traditional Korean and Chinese medicine [10]. Recent studies have also shown that the extract of each medicinal plant inhibits the proliferation of CRC cells [11,12,13,14,15,16]. Moreover, a basic theory of herbal combination in traditional medicine is the addition and subtraction theory that adds or/and removes one or more herbal medicines or dosages from an original foundational formula, thus generating another new formula for personalized medicine or better therapeutic effects [17]. Based on the information, we hypothesized that the addition of MCR to the main components of Paejangsan generating a new herbal formula might have anti-cancer properties for CRC. Therefore, we have in the present study developed a new herbal formula as an alternative to CRC treatments, which is comprised of PR (Paejang in Korean), MCR (Sangbekpi in Korean), and CS (Yiyiin in Korean), hence the name PSY, and have investigated whether this formula could be a potential therapeutic intervention for treating CRC.

## 2. Results and Discussion

### 2.1. PSY Synergistically Suppresses Cell Viability in Human CRC Cells

**Figure 1:**
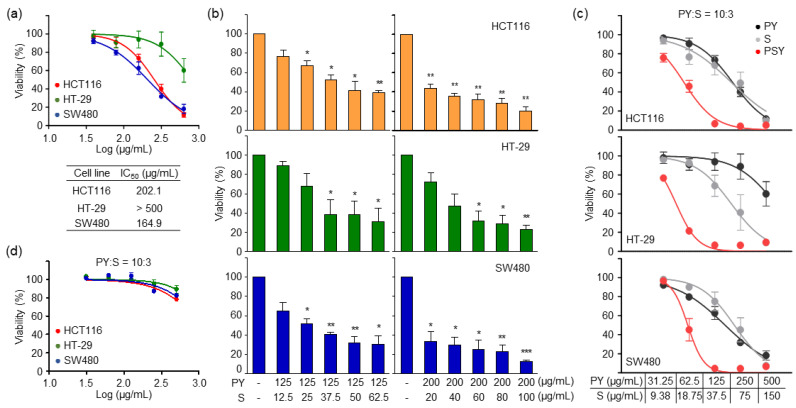
Effects of PSY extracts on the viability of human CRC cells. (**a**) Human CRC cells were treated with ethanol extract (EtOH-Ex) of Patriniae Radix and Coix Seed (PY) for 72 h as indicated. Cell viability was determined by MTT assays. IC_50_ values of PY EtOH-Ex in CRC cell lines were determined. (**b**) CRC cells were treated with five concentrations of Mori Cortex Radicis (S) EtOH-Ex in combination with PY EtOH-Ex (125 and 200 μg/mL) for 72 h. The ratio of PY and S was from 10-to-1 to 10-to-5. (**c**) CRC cells were treated with PSY EtOH-Ex in a 10-to-3 combination ratio and compared to each treatment of PY or S EtOH-Ex. (**d**) CRC cells were treated with PSY water extract (Water-Ex) in the combined ratio as indicated. Data are presented as the mean ± standard error of the mean (SEM) of results from at least three independent experiments performed in triplicates. *, *p* < 0.05, **, *p* < 0.01, ***, *p* < 0.001.

Since we aimed to develop PSY as a potential therapeutic intervention for CRC based on the addition and subtraction theory, we first examined whether PSY ethanol extract (EtOH-Ex) would synergistically affect the viability of human CRC cells compared to two main components of Paejangsan (PR and CS combination: PY) and MCR (S). Human CRC cells were treated with PY EtOH-Ex for 72 h, and cell viability was determined. As shown in Figure 1a, PY decreased cell viability in a dose-dependent manner in all CRC cell lines, but IC_50_ values in each cell line were quite high. In addition, the results indicated that HT-29 cells are quite resistant to PY (Figure 1a). We then performed the combination of IC_30_ (125 μg/mL) and IC_50_ (200 μg/mL) concentrations of PY in HCT116 with various concentrations of S EtOH-Ex to evaluate the potential effect of the combination and determine the best combination ratio. As shown in Figure 1b, an increase of S together with 125 μg/mL or 200 μg/mL of PY reduced cell viability in a dose-dependent manner in all CRC cell lines including HCT116. In addition, combinations of 37.5 μg/mL of S with 125 μg/mL of PY and 60 μg/mL of S with 200 μg/mL of PY were the lowest concentrations showing significant effects in all CRC cells including HT-29 (Figure 1b). These results suggested the combination of PY and S in a 10-to-3 ratio for the formula PSY. Additionally, the increase of PSY in the combination ratio (PY:S = 10:3) dramatically and synergistically suppressed the cell viability of all CRC cell lines tested compared to PY or S treatment (Figure 1c). However, PSY water extract (Water-Ex) did not show any significant effect (Figure 1d). These results indicated that PSY is potent to suppress the viability of CRC cells, and we further evaluated PSY EtOH-Ex in the combination ratio (PY:S = 10:3) as a potential anti-CRC agent.

### 2.2. PSY Induces Cell Cycle Arrest in the G_2_/M Phase and Apoptosis in Human CRC Cells

**Figure 2:**
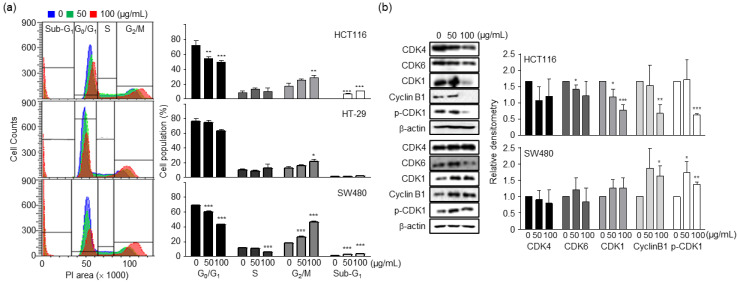
Effects of PSY on cell cycle in human CRC cells. CRC cells were treated with PSY EtOH-Ex for 48 h. (**a**) The treated cells were stained with PI, and the staining was analyzed using flow cytometry. The representative analysis is shown in the left panel. Every PI staining and analysis was performed at least three times in duplicate or triplicate. Cells were quantitated as a percentage of cells in each phase. Data in the right panel represent the mean ± SEM (*, *p* < 0.05; **, *p* < 0.01 and ***, *p* < 0.001 versus control). (**b**) Whole lysates of the treated cells were prepared, and Western blot analysis for CDK1, CDK4, CDK6, Cyclin B1, and phospho (p)-CDK1 was performed, and β-actin was used as an internal control. Data represent the mean ± standard deviation (SD) of three independent experiments (*, *p* < 0.05; **, *p* < 0.01 and ***, *p* < 0.001 versus control).

**Figure 3:**
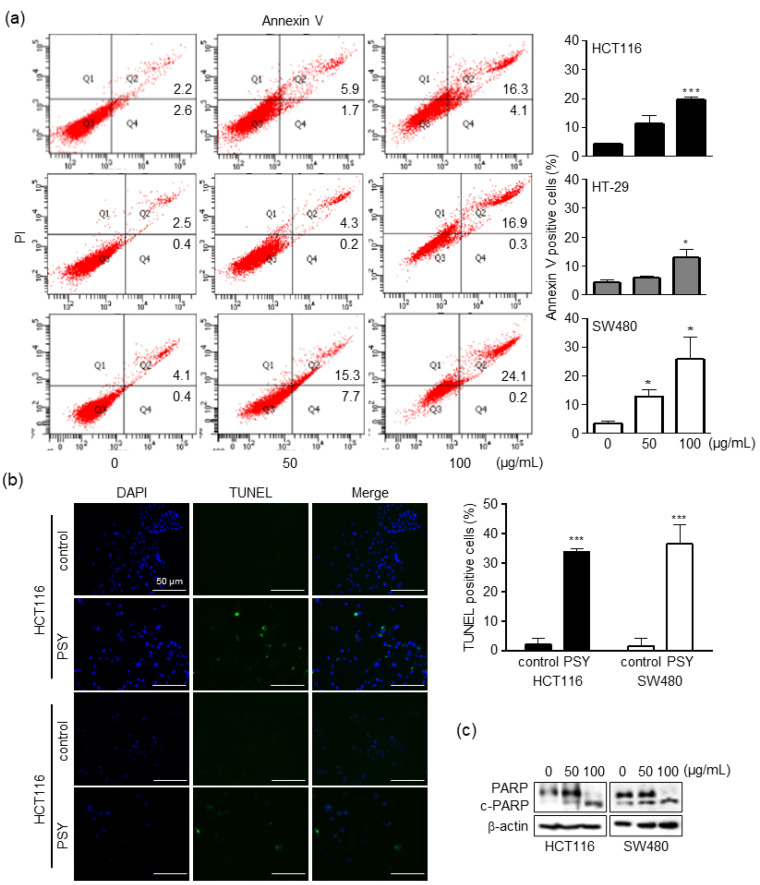
Effects of PSY on apoptosis in human CRC cells. CRC cells were treated with PSY EtOH-Ex for 48 h. (**a**) The cells were double-stained with FITC-Annexin V and PI and analyzed using flow cytometry (left panel). (**b**) The TUNEL and DAPI staining of the PSY (50 μg/mL)-treated cells were analyzed by confocal microscopy (left panel). Scale bar, 50 μm. Apoptotic cells were quantified as a percentage of Annexin V-positive and TUNEL-positive cells (right panel in **a**,**b**, respectively). Data present the mean ± SEM of three independent experiments (*, *p* < 0.05 and ***, *p* < 0.001 versus control). (**c**) Whole lysates of the treated cells were subjected to Western blotting for PARP and cleaved PARP (c-PARP). β-actin was used as an internal control.

As cell viability is regulated by cell proliferation and death, we investigated the effect of PSY on the cell cycle. As shown in Figure 2a, PSY increased the number of human CRC cells in the G_2_/M phase while gradually decreasing the number of cells in the G_0_/G_1_ phase. Accordingly, PSY decreased the expression levels of CDK1 and cyclin B1 in HCT116 cells and increased the phosphorylation of CDK1 in SW480 cells (Figure 2b), indicating that the inactivation of CDK1 leads to G_2_/M arrest. At the same time, PSY increased the CRC cell population in the sub-G_1_ phase (Figure 2a), suggesting the induction of apoptotic cell death. To further confirm whether PSY induces apoptosis, we stained PSY-treated CRC cells with annexin V-FITC and PI, and the proportion of apoptotic cells was determined. As shown in Figure 3a, PSY increased the number of annexin V-positive cells, indicating apoptosis. These results were also confirmed by terminal deoxynucleotidyl transferase dUTP nick end labeling (TUNEL) assays and examination of increased cleaved PARP, another apoptosis marker (Figure 3b,c). These results indicate that PSY decreases the viability of human CRC cells by inducing cell cycle arrest at the G_2_/M phase and apoptosis.

### 2.3. PSY Inhibits the STAT3 Pathway in Human CRC Cells

**Figure 4:**
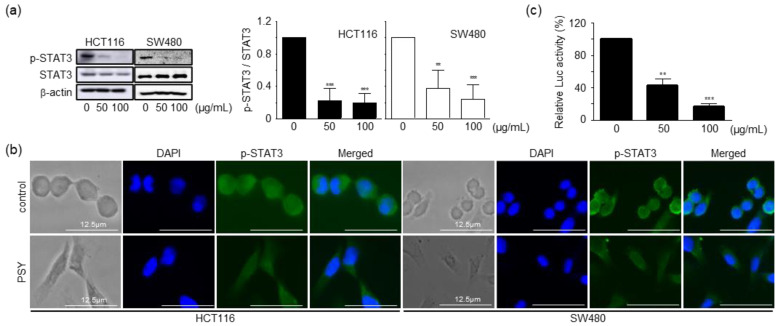
Effects of PSY on STAT3 in human CRC cells. (**a**) CRC cells were treated with PSY EtOH-Ex for 24 h, and cell lysates were subjected to Western blot analysis for STAT3 and p-STAT3. β-actin was used as an internal control. Data in the graphs represent the mean ± SD to that of the mock-treated cells taken as 1 (**, *p* < 0.01 and ***, *p* < 0.001 versus control). (**b**) Immunofluorescence staining of p-STAT3 and DAPI in the PSY (50 µg/mL)-treated CRC cells were analyzed by confocal microscopy. Scale bar, 12.5 μm. (**c**) HCT116 cells transfected with pSTAT3-Luc plasmid were treated with PSY for 24 h to analyze the transcriptional activity of STAT3. Data in the graphs are presented as the mean of fold-normalized luciferase (Luc) activities ± SEM to that of the untreated cells taken as 100% (**, *p* < 0.01 and ***, *p* < 0.001 versus control).

**Figure 5:**
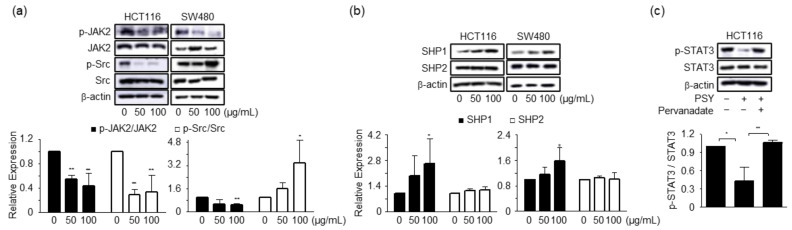
Effects of PSY on the STAT3 signaling pathway in human CRC cells. Whole lysates of CRC cells treated with PSY EtOH-Ex for 24 h were subjected to Western blot analysis for JAK2, p-JAK2, Src, and p-Src (**a**), and SHP1 and SHP2 (**b**). In addition, lysates of HCT116 cells treated with pervanadate (5 µM) and PSY (50 µg/mL) for 24 h were subjected to Western blot analysis for STAT3 and p-STAT3 (**c**). β-actin was used as an internal control. Data in graphs present the mean ± SD to that of the untreated cells taken as 1 (*, *p* < 0.05 and **, *p* < 0.01 versus control).

In the next step of the study, we attempted to determine the mechanism by which PSY induced cell cycle arrest and apoptosis in human CRC cells. The CRC development and progression are closely related to inflammation and the key molecule STAT3 [4]. Therefore, we prepared PSY with PR, MCR, and CS, each of which was traditionally used for treating inflammatory diseases. In this regard, we explored the effect of PSY on STAT3 expression. As shown in Figure 4a, PSY effectively reduced the phosphorylation of STAT3 in human CRC cells. PSY also inhibited the nuclear translocation of STAT3 (Figure 4b). Furthermore, PSY significantly decreased luciferase expression of the heterologous promoter system, which is regulated by STAT3 transcriptional activation in HCT116 cells (Figure 4c). STAT3 is constitutively activated in various types of human cancers and is associated with adverse clinical outcomes and poor prognosis in human CRC [18,19]. Recent studies have shown that PR and MCR induce apoptosis by inhibiting STAT3 in human multiple myeloma and CRC cells, respectively [15,16,20]. These results suggested that PSY induces apoptosis by inhibiting STAT3 in human CRC cells. Considering that JAK2, Src, and protein tyrosine phosphatases (PTPs), including SHP1 and SHP2, have been associated with STAT3 activation [6], we further examined the upstream signaling molecules of STAT3. As shown in Figure 5a, PSY decreased the phosphorylation of JAK2 and Src in HCT116 cells, while it only reduced the phosphorylation of JAK2 in SW480 cells. PSY also increased the expression of SHP1, a negative regulator of the JAK-STAT pathway, but not SHP2 in CRC cells (Figure 5b). In addition, pervanadate reversed PSY-mediated inhibition of STAT3 in HCT116 cells (Figure 5c), indicating a crucial role of SHP1 in the PSY mechanism of action. Accordingly, these results suggest that PSY suppresses human CRC cell proliferation by inducing cell cycle arrest and apoptosis via inhibiting the STAT3 signaling pathway.

### 2.4. Chemical Identification in PSY Extracts

**Figure 6:**
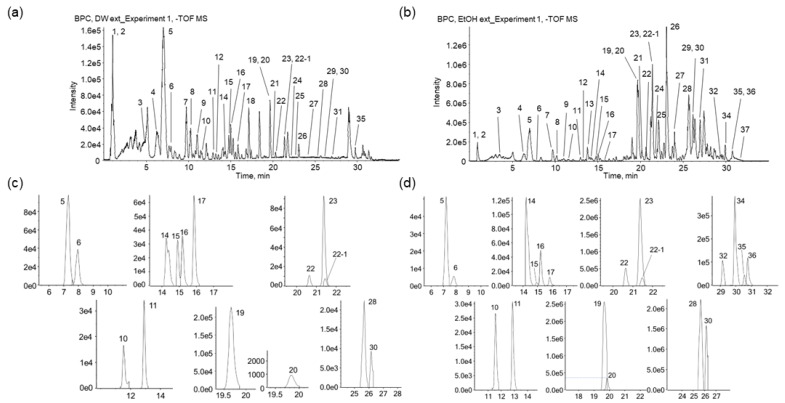
Chemical identification in PSY extracts. Representative BPCs of the Water-Ex (**a**) and EtOH-Ex (**b**) and expanded XICs from the Water-Ex (**c**) and EtOH-Ex (**d**) are presented. The scale of Y axis for intensity (CPS) on the chromatograms indicate that 1.6e5 equals 1.6 × 10^5^.

The chemical compositions of PSY EtOH-Ex and Water-Ex were characterized using UPLC-ESI-QTOF MS/MS in positive and negative ion modes. Representative base peak chromatograms (BPCs) of PSY Water-Ex and EtOH-Ex are shown in Figure 6a,b, and the identified minor or overlapping peaks on the BPCs are divided in the extracted-ion chromatograms (XICs) (Figure 6c,d). Detailed chemical and chromatographic information on the identified peaks (Figure 6) are summarized in Table 1, and their chemical profiles revealed 38 compounds of PSY, 7 organic acids, 8 iridoids, 2 lignans, 12 prenylflavonoids, 8 fatty acids, and 1 carbohydrate. 

Quinic acid (peak **2**) was identified using molecular networking (MN) analysis through Global Natural Products Social Molecular Networking (GNPS), and its derivatives, neochlorogenic acid (**3**), chlorogenic acid (**5**), cryptochlorogenic acid (**6**), 1,4-dicaffeoylquinic acid (**15**), 1,3-dicaffeoylquinic acid (**16**), and 4,5-dicaffeoylquinic acid (**17**), were identified by comparison with the retention time and fragmentation patterns of the reference standard in both positive and negative ion modes. Peaks **2**, **5**, **15**, **16**, and **17** have been reported as constituents of *P. scabiosaefolia* and *Partinia* [21,22].

**Figure 7:**
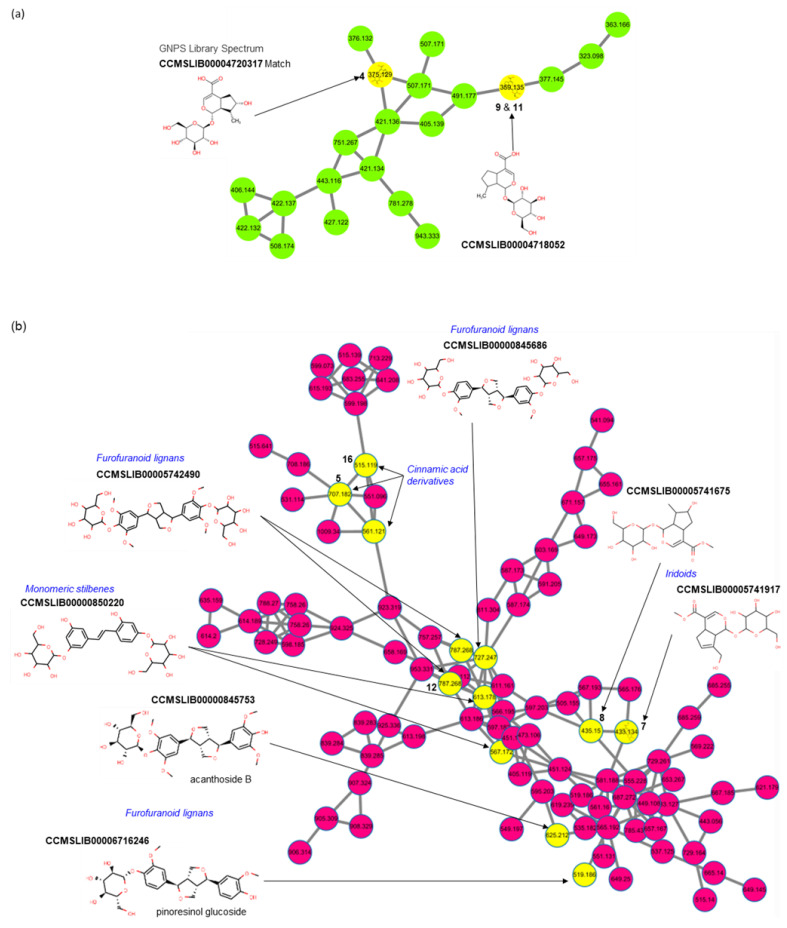

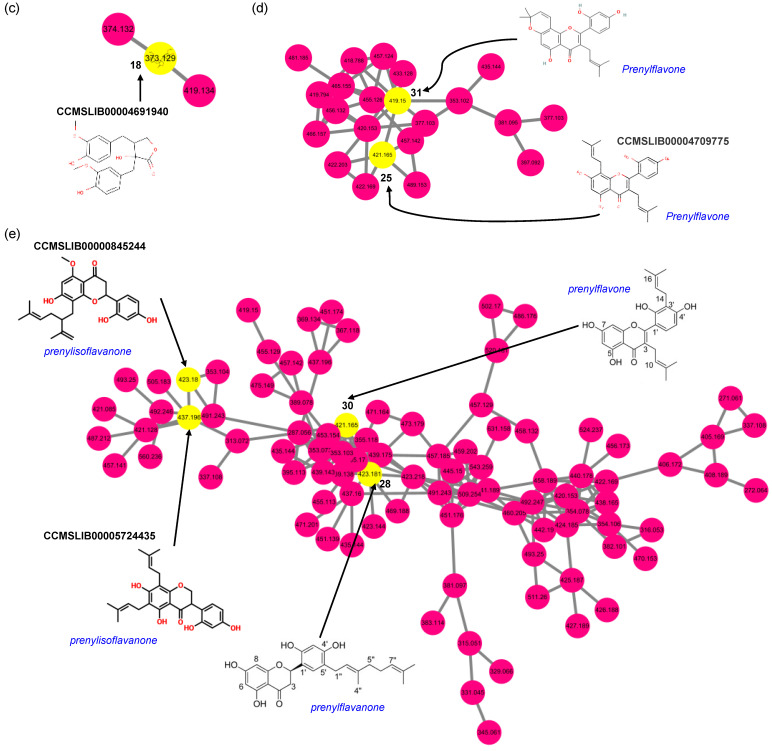
The representative MS/MS spectral network of identified phytochemical compounds in PSY extracts. Spectral nodes indicating identified compounds are noted with numbers (yellow nodes with peak numbers in Table 1). Annotated candidates for selected spectral nodes (yellow nodes without numbers) show compound name, GNPS library spectrum matching (in bold), and chemical class and subclass (in italic with blue). Molecular networks of representative chemical classes or biosynthetic pathways of natural products in PSY extracts. (**a**) iridoid class, (**b**) shikimate pathway including cinnamic (or quinic) acid derivatives, iridoid, furofuranoid lignan subclass in lignan, and stilbene class, (**c**) lignan class, (**d**) prenylflavone subclass in prenylflavonoid class, and (**e**) prenylisoflavanone, prenylflavanone, and prenylflavone subclasses in prenylflanoid class. Further information can be found at the GNPS job link (https://gnps.ucsd.edu/ProteoSAFe/status.jsp?task=e4406e69e619467b9a347a905d6c383a accessed on 31 March 2022).

Eight iridoids were identified in the PSY extracts. Loganic acid (**4**) and loganin (**8**) were identified using the reference standard and MN analysis on the GNPS. The loganic acid yielded its quasi-molecular ion [M−H]^−^ at *m*/*z* 375.1287 (mass error = −1.72), and its fragment ions were *m*/*z* 213.0762 [M−H−Glc]^−^, 169.0864 [M−H−Glc−CO_2_], 151.0757 [M−H−Glc−CO_2_−H_2_O], 113.0243 [M−H−Glc−C_3_O_4_], and 69.0369 (C_5_H_9_, isoprenyl moiety). The precursor ion at *m*/*z* 359.1344 was putatively identified as a deoxyloganic acid isomer (**9** and **11**) in MN analysis (Figure 7a). The mass difference (15.9943 Da, oxygen) of the precursor ion between loganic acid and deoxyloganic acid was found at *m*/*z* 197.0823, 135.0823, and 153.0915 from the fragment ions of **9** and **11**, respectively. The precursor ions of deoxyloganic acid were detected at peaks **9** (RT 11.1 min) and **11** (RT 12.9 min) on BPC and XIC (Figure 6). Although compounds **9** and **11** were identified as deoxyloganic acid isomers, they could not be clearly classified as 7-deoxyloganic acid or 8-epideoxyloganic acid. Geniposide (**7**) was putatively identified through MN analysis on the GNPS (Figure 7b). Patrinalloside (**10**) and patrinoside isomers (**13** and **14**) have been identified in the literature [23,24,25,26]. Some iridoids (**4**, **8**, **9**, **10**, and **11**) are representative components of *P. scabiosaefolia* [21,22]. 

Eleutheroside E (**12**) and nortrachelogenin (**18**) were detected and putatively identified as lignans by matching the MS/MS fragment ions with the MN analysis on the GNPS (Figure 7b,c). Nortrachelogenin has been previously isolated from PR [27].

Twelve prenylflavonoids originating from MCR were detected, and **23** and **31** were identified as kuwanon G and morusin [28,29], representative components in MCR with each reference standard, respectively. Peak **23** is the Diels-Alder (DA)-type adduct of a chalcone and prenylflavone and exhibited an [M−H]^−^ ion at *m*/*z* 691.2182. In negative ion mode, fragment ions were observed at *m*/*z* 581.1820 [M−H−resorcinol (C_6_H_6_O_2_, 110 Da)]^−^, 353.1029 [M−H−resorcinol−C_14_H12O_3_]^−^, 419.1501, 379.1189 [M-H-2resorcinol−C_4_H_8_O−H_2_O]^−^, and 539.1719 [M−H−resorcinol−C_3_H_6_]^−^. Morusin exhibited characteristic fragment ions at *m*/*z* 297.1134, 191.0716, 309.1139 [M−H−resorcinol]^−^, and 350.04804 [M−H−isoprenyl]^−^. The fragment ion at *m*/*z* 191.0716 lost one CO and received two hydrogens at C-10 from *m*/*z* 217.0507, produced by retro-DA cleavage [30]. Peak **25** was detected and putatively identified as Kuwanon C through MN analysis and clustered with **31** as prenylflavones (Figure 7d). The mass difference (2 Da, two hydrogens) between the precursor ions of peaks **31** and **25** was found in the characteristic fragmentation patterns (Table 1). Peaks **28** and **30** were putatively identified as Kuwanon E and Kuwanon T using PeakView 2.2, using exact mass and isotope patterns. Kuwanon E was further identified by fragmentation patterns in FooDB. Additionally, **28** and **30** (prenylflavones) were clustered by MN analysis, with two isoflavanones (not shown in Table 1) identified by library matching (Figure 7e). Mulberrofuran G, kuwanon G, kuwanon T, sanggenon F, and morusin, found in MCR, are known to exert anti-inflammatory and anti-cancer activities [15,31]. Kuwanon G also ameliorates lipopolysaccharide-induced disruption of the gut epithelial barrier [32,33]. In addition, morusin induces autophagy and apoptosis by regulating various signaling molecules, including STAT3, in several types of cancer cells [31,32].

Peaks **27**, **29**, and **32**–**37** were identified as fatty acids (Table 1), and linoleic (**34**) and oleic (**37**) acids are known to be present in CS [34]. Moreover, Linolenic, linoleic, and oleic acids are known to have various pharmacological activities, including anti-inflammatory, anti-cancer, and anti-microbial activities [35,36].

**Table 1:** Identification of chemical components in PSY using LC-ESI-QTOF MS/MS.

Peak No.	Name	Formula	Mass (Da)	Expected RT (min)	Adduct	Detected at Mass (Da)	Error (ppm)	Fragment Ions (MS/MS Product Ions)	Identified with	Chemical Class
1	Sucrose	C_12_H_22_O_11_	342.1162	0.9	[M−H] ^–^	341.1091	0.3	89.0258, 179.0565, 119.0359, 59.0166, 71.0166, 113.0253	# GNP	*Carbohydrate*
2	Quinic acid	C_7_H_12_O_6_	192.0634	0.9	[M−H] ^–^	191.0567	0.8	85.0315, 93.0362	# in-house & GNP	*Quinic acid derivatives*
3	Neochlorogenic acid (3-O-caffeoylquinic acid; 3-CQA)	C_16_H_18_O_9_	354.0951	4.5	[M+H] ^+^	355.1021	−0.6	163.0373, 145.0284, 135.0438, 117.0326	†	*Quinic acid derivatives*
					[M−H] ^–^	353.0877	−0.3	191.0564, 179.0345, 135.0462, 134.0372		*Quinic acid derivatives*
4	Loganic acid	C_16_H_24_O_10_	376.1370	6.6	[M−H] ^−^	375.1287	−1.7	213.0762, 169.0864, 151. 0757, 113.0243, 69.0369	†	*Iridoids*
5	Chlorogenic acid (5-O-caffeoylquinic acid; 5-CQA)	C_16_H_18_O_9_	354.0951	7.3	[M+H] ^+^	355.1025	0.3	163.0388, 145.0283, 135.0439, 117.0333	†	*Quinic acid derivatives*
					[M−H] ^–^	353.0871	−2.1	191.0566, 161.0252, 173.0464		*Quinic acid derivatives*
6	Cryptochlorogenic acid (4-O-caffeoylquinic acid; 4-CQA)	C_16_H_18_O_9_	354.0951	7.9	[M+H] ^+^	355.1024	0.2	163.0390, 145.0284, 135.0442, 117.0343	†	*Quinic acid derivatives*
					[M−H] ^–^	353.0874	−1.1	191.0557, 173.0449, 135.0451, 179.0350		*Quinic acid derivatives*
7	Geniposide	C_17_H_24_O_10_	388.1370	9.7	[M−H+FA] ^–^	433.1341	0.2	225.0772, 387.1270, 101.0255, 123.0455	# GNP	*Iridoids*
8	Loganin	C_17_H_26_O_10_	390.1526	10.2	[M−H+FA] ^–^	435.1498	0.3	227.0929, 127.0414, 101.0264, 389.1474	†	*Iridoids*
9	Deoxyloganic acid (7- or 8-epi)	C_16_H_24_O_9_	360.1420	11.1	[M−H] ^–^	359.1344	−2.0	197.0823, 135.0823, 153.0915	# FooDB, GNP & Ref. [21]	*Iridoids*
10	Patrinalloside	C_21_H_34_O_11_	462.2101	11.6	[M−H+FA] ^–^	507.2070	−0.9	361.1498, 403.1605, 343.1391, 161.0449	Refs. [25,26]	*Iridoids*
11	Deoxyloganic acid (7- or 8-epi)	C_16_H_24_O_9_	360.1420	12.9	[M−H] ^–^	359.1344	−1.1	197.0818, 135.0817, 153.0916,59.0167	# FooDB, GNP & Ref. [21]	*Iridoids*
12	Eleutheroside E	C_34_H_46_O_18_	742.2684	13.4	[M−H+FA] ^–^	787.2670	1.8	579.2089, 417.1554, 181.0506	# GNP	*lignans*
13	Patrinoside/isomers	C_21_H_34_O_11_	462.2101	13.8	[M−H+FA] ^–^	507.2070	−0.4	179.0564, 89.0260, 119.0356, 161.0459, 377.1444	Refs. [23,24]	*Iridoids*
14	Patrinoside/isomers	C_21_H_34_O_11_	462.2101	14.3	[M−H+FA] ^–^	507.2070	−0.1	179.0559, 461.2007, 89.0262, 377.1447, 119.0358, 161.0457	Refs. [23,24]	*Iridoids*
15	1,4-Dicaffeoylquinic acid	C_25_H_24_O_12_	516.1268	14.9	[M+H] ^+^	517.1316	−2.4	163.0377, 145.0273, 499.1216, 319.0789	†	*Quinic acid derivatives*
					[M−H] ^–^	515.1183	−2.3	353.0869, 179.0340, 173.0452, 191.0553		
16	1,3-Dicaffeoylquinic acid	C_25_H_24_O_12_	516.1268	15.2	[M+H] ^+^	517.1332	−1.7	163.0391, 145.0294, 135.0431, 117.0331	†	*Quinic acid derivatives*
					[M−H] ^–^	515.1188	−1.4	353.0866, 191.0556, 179.0338, 135.0455		
17	4,5-Dicaffeoyl quinic acid	C_25_H_24_O_12_	516.1268	15.9	[M+H] ^+^	517.1338	−0.6	163.0382, 145.0260, 135.0421,337.0906	†	*Quinic acid derivatives*
					[M−H] ^–^	515.1191	−0.8	353.0874, 173.0454, 179.0346, 191.0556		
18	Nortrachelogenin	C_20_H_22_O_7_	374.1366	17.1	[M−H] ^–^	373.1291	−0.5	179.0714, 164.0476, 99.0099, 327.1238, 163.0476	# GNP	*lignans*
19	Kuwanon L	C_35_H_30_O_11_	626.1788	19.7	[M+H] ^+^	627.1853	−1.2	309.1740, 153.0181, 137.0233, 433.0908, 499.1374	*	*Diels-Alder (DA)-type flavonoids*
					[M−H] ^–^	625.1716	0.1	499.1406, 389.1025, 125.0253, 109.0306		
20	Kuwanon Y	C_34_H_30_O_9_	582.1890	19.8	[M+H] ^+^	583.1947	−2.6	203.0706, 137.0237, 339.1218, 473.1585	# FooDB	*Diels-Alder (DA)-type flavonoids*
					[M−H] ^–^	581.1813	−0.6	361.1089, 471.1463, 227.0716, 243.0666, 563.1738		
21	Mulberrofuran G	C_34_H_26_O_8_	562.1628	20.2	[M+H] ^+^	563.1695	−0.9	441.1345, 255.0661, 123.0449, 387.0873	*	*Diels-Alder (DA)-type flavonoids*
					[M−H] ^–^	561.1540	−1.1	451.12190, 439.1187, 433.1072, 241.0508	*	
22	Sangenon F/isomers	C_20_H_18_O_6_	354.1103	20.6	[M+H] ^+^	355.1178	0.4	153.0189	*	*Prenylflavonoids*
					[M−H] ^–^	353.1027	−1.0	227.0712, 125.0251, 201.0920		
23	Kuwanon G	C_40_H_36_O_11_	692.2258	21.4	[M+H] ^+^	693.2321	−1.4	137.0235, 203.0708, 365.1026, 299.0556, 421.1654	†	*Diels-Alder (DA)-type flavonoids*
					[M−H] ^–^	691.2182	−1.0	581.1820, 353.1029, 419.1501, 379.1189, 539.1719		
22-1	Sangenon F/ isomers	C_20_H_18_O_6_	354.1103	21.5	[M+H] ^+^	355.1178	0.7	153.0184	*	*Prenylflavonoids*
					[M−H] ^–^	353.1027	−0.8	125.0257, 201.0927, 227.0712		
24	Kuwanon O or Sanggenon G/isomers	C_40_H_38_O_11_	694.2374	22.1	[M+H] ^+^	695.2452	−2.2	205.0858, 341.1381, 267.0650, 677.2367	Ref. [29]	*Diels-Alder (DA)-type flavonoids*
			694.2433	22.1	[M−H] ^–^	693.2350	−0.7	567.2098, 389.1034, 177.0919, 125.0243		
25	Kuwanon C	C_25_H_26_O_6_	422.1729	22.8	[M+H] ^+^	423.1802	−1.8	311.0549, 241.0494, 283.0599, 367.1179	# GNP	*Prenylflavonoids*
					[M−H] ^–^	421.1654	−0.7	299.1292, 309.0411, 193.0870, 219.0667, 297.0408		
26	Kuwanon O/isomer	C_40_H_38_O_11_	694.2374	23.1	[M+H] ^+^	695.2452	0.7	271.1323, 137.0234, 407.1849, 297.1482	*	*Diels-Alder (DA)-type flavonoids*
			694.2433	23.1	[M−H] ^–^	693.2350	−1.5	531.2031, 287.0563, 259.1341, 125.0253, 583.1993	*	
27	13-keto-9Z,11E-octadecadienoic acid	C_18_H_30_O_3_	294.2195	24.1	[M−H] ^–^	293.2120	−0.6	275.2007, 235.1692, 183.1383, 171.1018	*	*Fatty acids*
28	Kuwanon E	C_25_H_28_O_6_	424.1886	25.7	[M+H] ^+^	425.1955	−0.8	153.0183, 175.3939	# FooDB	*Prenylflavonoids*
					[M−H] ^–^	423.1806	−1.7	297.1492, 125.0250, 271.1705, 177.0195, 245.1545, 405.1703		
29	13-Hydroxyoctadecadienoic acid	C_18_H_32_O_3_	296.2352	26.0	[M−H] ^–^	295.2272	0.0	277.2181, 195.1392, 171.1033	*	*Fatty acids*
30	Kuwanon T	C_25_H_26_O_6_	422.1729	26.2	[M+H] ^+^	423.1796	−1.5	153.0178, 175.0385	*	*Prenylflavonoids*
					[M−H] ^–^	421.1657	0.1	295.1341, 125.0252, 269.1550, 174.0329, 151.0040		
31	Morusin	C_25_H_24_O_6_	420.1573	27.0	[M+H] ^+^	421.1645	−0.3	365.1012, 347.0913, 332.0682, 295.0953	†	*Prenylflavonoids*
				27.0	[M−H] ^–^	419.1501	0.2	297.1134, 191.0716, 309.1139, 350.04804, 217.0507		
32	Linolenic acid	C_18_H_30_O_2_	278.2246	29.2	[M−H] ^–^	277.2175	0.8	277.2175	# GNP	*Fatty acids*
33	Oleamide	C_18_H_35_NO	281.2719	29.8	[M+H] ^+^	282.2794	0.9	247.2419, 265.2525, 97.1019, 135.1168, 121.1013,149.1321	# GNP	*Fatty acid amide*
34	Linoleic acid	C_18_H_32_O_2_	280.2402	29.9	[M−H] ^–^	279.2335	1.9	279.2335	†	*Fatty acids*
35	3-Oxo-olean-12-en-28-oic acid	C_30_H_46_O_3_	454.3447	30.4	[M−H] ^–^	453.3365	−2.1	453.3365	†	*Fatty acids*
36	Palmitic acid	C_16_H_32_O_2_	256.2402	30.5	[M−H] ^–^	255.2329	−0.4	255.2329	†	*Fatty acids*
37	Oleic acid	C_18_H_34_O_2_	282.2559	31.7	[M−H] ^–^	281.2485	−0.6	283.2643	†	*Fatty acids*

# In-house ms/ms library and online database; such as GNPS, MASS bank or Metlin; † Reference standard; and * Extract MS with isotope mass.

### 2.5. Identification of Potential Bioactive Phytochemicals in PSY EtOH-Ex

**Figure 8:**
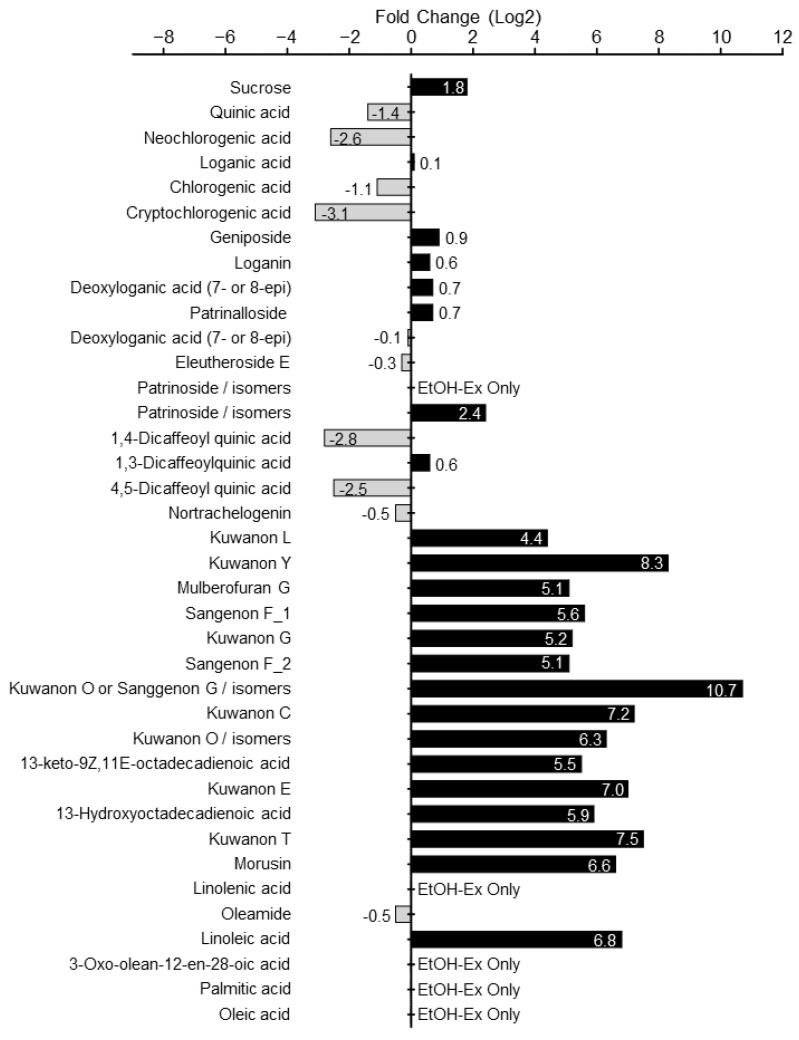
Identification of PSY EtOH-Ex-enriched phytochemicals. Peak area fold change of 38 compounds from PSY EtOH-Ex and Water-Ex. Fold change > 0 means that the relative content of chemical compounds in EtOH-Ex is higher than Water-Ex. The fold changes in peak area were calculated using the formula log2 for the ratio of peak area values of the phytochemicals extracted by different solvents.

To investigate the potential bioactive candidates in EtOH-Ex, due to its potent anti-cancer activity compared to that of Water-Ex, the fold change in the peak area of every compound in EtOH-Ex versus Water-Ex was calculated and compared because the fold change reflects the degree of differences in chemical concentration. As shown in Figure 8, the comparison of ploidy area variation was significant for 21 phytochemicals (fold change > 2), including 12 prenylflavonoids, two iridoids, and seven fatty acids. All prenylflavonoids were significantly enriched in EtOH-Ex. Intriguingly, four fatty acids (**32**, **35**, **36**, and **37**) and one iridoid (**13**) were found only in the EtOH-Ex. These results suggest that the EtOH-Ex-enriched compounds could be potential bioactive molecules, indicating the PSY effect to suppress CRC cell proliferation, and could be a useful source for developing anti-cancer drugs. Additionally, other phytochemicals remain unknown. Since these candidates may also contribute to anti-cancer effects, further identification of novel compounds, evaluation of their anti-cancer effects, and further investigation of their mechanisms of action seem to be necessary.

Although many early stage CRC patients undergo surgery such as colectomy or proctectomy without chemotherapy, most patients, especially those suffering from metastatic CRC require chemotherapy with or without surgery and radiotherapy [37]. Conventional combinatorial strategies, FOLFOX (folinic acid, 5-fluorouracil (5-FU), and oxaliplatin) or FOLFIRI (folinic acid, 5-FU, and irinotecan) have been the most appropriate first-line chemotherapy options for CRC patients since their development in the early 2000s [38,39]. The recent addition of molecular-targeted drugs such as cetuximab and encorafenib has been a major improvement with more effective therapeutic options [39]. These therapeutic advances have resulted in clinically relevant survival improvements, but the five-year survival rate is still less than 15% for stage IV disease and most survivors suffer from severe side effects including neuropathy, bowel and bladder dysfunction, and sexual dysfunction [1,38,39]. Therefore, novel therapeutic strategies are still required for this fatal disease. We, thus, have focused on medicinal plants which have been traditionally used for thousands of years and are edible, expecting at least fewer adverse effects, and found the combination of PSY in the present study.

**Figure 9:**
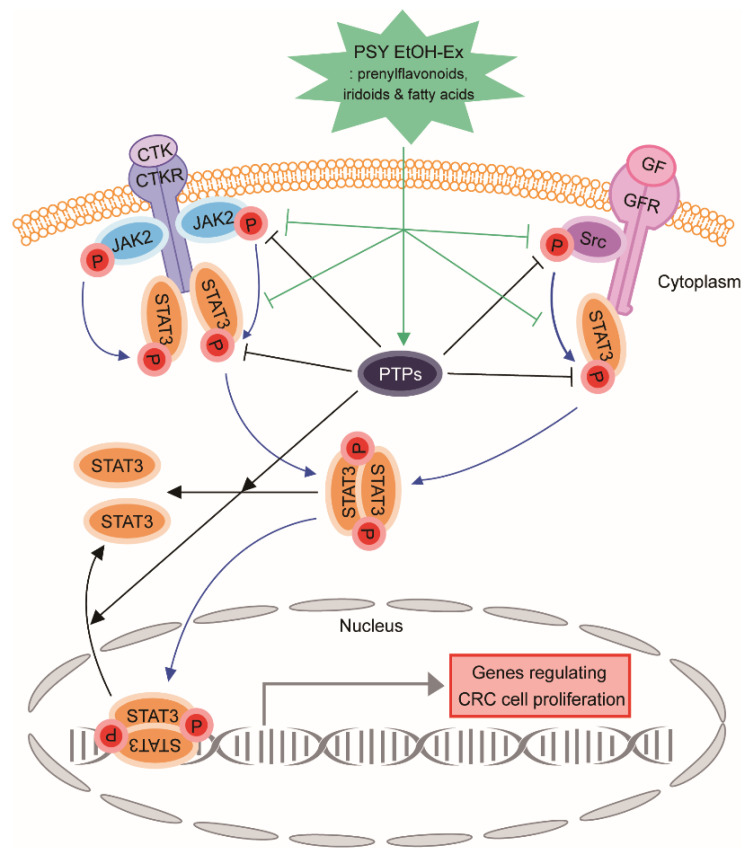
A schematic representation of the molecular mechanism by which PSY suppresses human CRC cell proliferation. CTK: cytokine, CTKR: cytokine receptor, GF: growth factor, GFR: growth factor receptor, PTP: protein tyrosine phosphatase, CRC: colorectal cancer.

Altogether, these results demonstrate that PSY likely suppresses CRC cell proliferation by inducing cell cycle arrest and apoptosis via inhibiting oncogenic STAT3 signals. Further, we found 21 EtOH-Ex-enriched PSY phytochemicals, suggesting further studies investigating the potential anti-cancer activities of these compounds and their mechanisms of action involved in STAT3 (Figure 9). In conclusion, these results suggest that PSY could be a potentially effective therapeutic for patients with CRC. More research about the potential combination of PSY or an EtOH-Ex-enriched PSY phytochemical with any conventional drug including 5-FU, oxaliplatin, and irinotecan would be also interesting and facilitate the development of a more effective and therapeutic strategy with less adverse effects for CRC patients.

## 3. Materials and Methods

### 3.1. PSY Preparation

Dried PR, MCR, and CS were purchased from Omniherb (Daegu, Republic of Korea), regulated in herbal Good Manufacturing Practices (hGMP), and each voucher specimen was deposited in the herbarium of the Korean Medicine Clinical Trial Center, Kyung Hee University Korean Medicine Hospital (Seoul, Republic of Korea). For EtOH-Ex preparation, 37.5 g of PR, 22.5 g of MCR, and 37.5 g of CS were extracted with 877.5 mL of ethanol by refluxing at room temperature sonication. Similarly, for the preparation of PSY Water-Ex, the amount of PR, MCR, and CS was extracted with 877.5 mL of distilled water by refluxing at 100 °C for 1.5 h. Each extract was filtered through vacuum filter paper and concentrated in a rotary evaporator (Eyela N-1200 system, Tokyo, Japan) at 40 °C under lower pressure. The extracts were lyophilized, and the yields of dried EtOH-Ex and Water-Ex were 3.21% and 10.13%, respectively. Each extract was dissolved in dimethyl sulfoxide (DMSO) to obtain a 500 mg/mL stock solution.

### 3.2. Chemicals and Reagents

Standard references, including neochlorogenic acid, loganic acid, cryptochlorogenic acid, loganin, kuwanon G, and 3-Oxo-olean-12-en-28-oic acid, were purchased from ChemFaces (Wuhan, China). Linoleic and palmitic acids were obtained from Sigma-Aldrich (St. Louis, MO, USA). Morusin and chlorogenic acid were obtained from Biopurity Phytochemicals, Ltd. (Chengdu, China). Further, mixed references were prepared by dissolving the references in a 50% methanol in water (*v*/*v*) solution for qualitative analysis using UPLC-ESI-QTOF MS/MS.

### 3.3. Cell Lines and Culture Condition

HCT116, HT-29, and SW480 human CRC cell lines were acquired from the Korean Cell Line Bank (Seoul, Republic of Korea) and were cultured according to the manufacturer’s instructions.

### 3.4. Cell Viability Assays

CRC cells (3–5 × 10^3^ cells/well) were seeded in 96-well plates and incubated overnight. Then, the cells were treated with PSY for 72 h. After the treatment, cell viability was measured by 3-(4,5-dimethylthiazol-2-yl)-2,5-diphenyl-tetrazolium bromide (MTT) assays as previously described [40]. After dissolving formazan crystals with DMSO, the absorbance was monitored at 570 nm using a microplate reader (Molecular Devices, CA, USA Data are presented as standard error of means (SEM) from at least three independent experiments in triplicate.

### 3.5. Cell Cycle Analysis

CRC cells (2–3 × 10^5^ cells/well) in 6-well plates were treated with PSY for 48 h. Then, the cells were harvested by trypsinization, rinsed twice with cold PBS, and pelleted by centrifugation at 1200 rpm for 3 min. Pellets were re-suspended in 70% ice-cold EtOH for 30 min at 4 °C to perform cell fixation. Then, samples were rinsed twice with PBS and stained using a solution containing 100 μg/mL of RNase A, 50 μg/mL propidium iodide (PI) in PBS for 30 min in the dark ([31]). Then, the cell cycle of the treated cells was analyzed using a BD FACS Canto™ II (Franklin Lakes, NJ, USA).

### 3.6. Apoptosis Analysis

Apoptosis of CRC cells was analyzed using a fluorescein isothiocyanate (FITC) Annexin V apoptosis detection kit (Biovision, Inc. K101-100, Waltham, MA, USA). Cells (2–3 × 10^5^ cells/well) in 6-well plates were treated with PSY for 48 h. After the treatment, both adherent and floating cells were harvested by centrifugation, washed with PBS, and resuspended in 1× binding buffer at 1 × 10^6^ cells/mL. Then, 100 μL of the cell suspension was transferred to a 5 mL culture tube and incubated with 5 μL FITC Annexin V and 5 μL PI for 15 min at room temperature in the dark. Then, 400 μL of 1× binding buffer was added in the 5 mL culture tube. Fluorescence intensity was analyzed using a BD FACS canto II (Franklin Lakes, NJ, USA). In addition, TUNEL assays were performed using a DeadEnd™ Fluorometric TUNEL assay kit (Promega, Madison, WI, USA) according to the manufacturer’s protocol.

### 3.7. Immunofluorescence Staining

Cells were seeded in 8-well chamber slides and treated with PSY for 24 h. After the cells were fixed and permeabilized, the cells were stained with rabbit polyclonal anti-phospho (p)-STAT3 (1:100), Alexa Flour 488 goat anti-rabbit antibody (1:1000) (Invitrogen, Carlsbad, CA, USA) and 4′,6-diamidino-2-phenylindole (DAPI) (Sigma-Aldrich) as described ([31]. Images were obtained using an iRiSTM Digital Cell Imaging System (Logos Biosystems, Anyang, Republic of Korea).

### 3.8. Transient Transfection and Luciferase (Luc) Assays

Transient transfection and Luc assays were performed to measure STAT3 activity in PSY-treated cells as previously described ([31]). Cells were transfected with pSTAT3-Luc reporter containing STAT3 binding sites to measure STAT3 activity (Clontech, Palo Alto, CA, USA) in a 60 mm plate using Lipofectamine 2000 (Invitrogen). One day after transfection, cells were re-plated into a 48-well plate. Then, the cells were treated with PSY for 24 h. Luc assays were performed using a Luc assay system (Promega, Madison, WI, USA) according to the manufacturer’s instructions. Luc activity was normalized by protein concentration.

### 3.9. Western Blotting Analysis

Whole-cell lysates were prepared, and Western blotting was performed as previously described ([31]). Primary antibodies against STAT3, p-STAT3 (Tyr705), JAK2, Src, p-Src (Tyr416), CDK4, CDK6, and p-CDK1 (Tyr15) were purchased from Cell Signaling Technology (Danvers, MA, USA). Additionally, p-JAK2 (Tyr1007/1008), CDK1, cyclin B1, PARP, SHP1, SHP2, and β-actin were purchased from Santa Cruz Biotechnology (Santa Cruz, CA, USA), and HRP-conjugated anti-mouse IgG and anti-rabbit (Cell Signaling Biotechnology) were used. Densitometric analysis of each band was performed using the ImageJ software (https://imagej.nih.gov/ij accessed and downloaded the updated software on 27 September 2021). Data are presented as the mean ± standard deviation (SD) from at least three independent experiments.

### 3.10. Ultra-Performance Liquid Chromatography Quadrupole Time of Flight Mass Spectrophotometry (UPLC-ESI-QTOF MS/MS) Analysis

Chromatographic analysis of the extract was performed to identify and assess the quality of the chemical components of the EtOH-Ex and Water-Ex. The LC-MS/MS system consisted of a Thermo Scientific Vanquish UHPLC system (Sunnyvale, CA, USA) with an ACQUITY UPLC HSS T3 column (2.1 mm × 100 mm, 1.8 μm; Waters^TM,^, Milford, MA, USA) and a Triple TOF 5600+ MS system (QTOF MS/MS, SCIEX, Foster City, CA, USA). The QTOF MS was equipped with an ESI source and used to complete the high-resolution experiment.

### 3.11. Phytochemical Identification in PSY

Raw data detected by UPLC-QTOF MS/MS were analyzed using PeakView 2.2, including MasterView software (SCIEX) for peak identification and matching with the reference standard and in-house MS/MS library, and the chemical structural information was analyzed using the GNPS website (http://gnps.ucsd.edu, accessed on 31 March 2022). Subsequently, accurate MS/MS fragmentation information was queried in MassBank (https://massbank.eu/MassBank), FooDB (https://foodb.ca), and Metlin databases (http://metlin.scripps.edu/website) to screen and identify potential compound information.

### 3.12. Statistical Analysis

All experiments were performed at least thrice. Data were analyzed using GraphPad Prism software (version 5.03; GraphPad Software, Inc., La Jolla, CA, USA) and expressed as mean ± SEM or SD. Differences between groups were assessed using analysis of variance, followed by ANOVA-Tukey’s post hoc test, and *p* < 0.05 was considered to indicate a statistically significant difference.

## Data Availability

Not applicable.
